# Resveratrol and STAT inhibitor enhance autophagy in ovarian cancer cells

**DOI:** 10.1038/cddiscovery.2015.71

**Published:** 2016-01-25

**Authors:** L-X Zhong, Y Zhang, M-L Wu, Y-N Liu, P Zhang, X-Y Chen, Q-Y Kong, J Liu, H Li

**Affiliations:** 1 Department of Clinical Oncology, Second Affiliated Hospital of Dalian Medical University, Dalian 110042, China; 2 Liaoning Laboratory of Cancer Genetics and Epigenetics, College of Basic Medical Sciences, Dalian Medical University, Dalian 116044, China; 3 School of Medicine, South China University of Technology, Guangzhou 510006, China

## Abstract

Autophagic activity reflects cellular response to drug treatment and can be regulated by STAT3 signaling. Resveratrol inhibits STAT3 activation and causes remarkable growth arrest and cell death of ovarian cancer (OC) cells. However, the autophagic status and its relevance with resveratrol’s anti-OC effects remain unclear. We analyzed the states of autophagic activities, the nature of autophagosomes and the levels of autophagy-related proteins (LC-3, Beclin 1 and STAT3) in resveratrol-treated CAOV-3 and OVCAR-3 OC cells using multiple approaches. We elucidated the correlation of STAT3 inhibition with autophagic activity by treating OC cells with an upstream inhibitor of STAT proteins, AG490. Resveratrol efficiently suppressed growth, induced apoptosis and inactivated STAT3 signaling of the two OC cell lines. We found enhanced autophagic activity accompanied with Beclin-1 upregulation and LC3 enzymatic cleavage in resveratrol-treated OC cells. Immunofluorescent (IF) microscopic and IF-based confocal examinations demonstrated the accumulation of cytoplasmic granules co-labeled with LC3 and cytochrome C in resveratrol- or AG490-treated OC cells. Using electron microscopy, we confirmed an increase in autophagosomes and mitochondrial spheroids in either resveratrol- or AG490-treated OC cells. This study demonstrates the abilities of resveratrol to enhance apoptotic and autophagic activities in OC cells, presumably via inactivating STAT3 signaling. Resveratrol or the selective JAK2 inhibitor also leads to mitochondrial turnover, which would be unfavorable for OC cell survival and sensitize OC cells to resveratrol.

## Introduction

Ovarian cancer (OC) is one of the commonest female malignancies with an extremely poor prognosis.^1–3^ Surgical treatment is the first choice for removing OCs in cases that are well-differentiated, relatively small, or confined to the ovary.^4,5^ Unfortunately, the majority of OC patients (75%) are diagnosed at advanced stages because of the subtle symptoms at the early stages of ovarian carcinogenesis.^6^ Consequently, most OC patients die of metastases due to peritoneal transplantation or blood stream spreading.^7^ Therefore, adjuvant chemotherapy is required to prevent tumor relapse and dissemination.^8^

Although more accurate staging of the disease and more aggressive surgical excision of tumor spots in the abdomen have somewhat improved therapeutic outcomes, the overall survival rates continue to lack promise.^9^ Furthermore, drug resistance often occurs among OC patients and severe toxic effects caused by conventional anticancer drugs greatly reduce patients’ quality of life.^9–11^ It is therefore urgent to explore more effective and less toxic agents with clearer molecular targets for better adjuvant management of OCs.

It has been increasingly recognized that resveratrol (3,5,4′-trihydroxy-*trans*-stilbene) possesses cancer-preventive and -suppressive activities.^12–14^ More importantly, resveratrol has little cytotoxic effect on normal tissues *in vitro* and *in vivo* at effective anticancer doses, reflecting its potential value in cancer treatments when administered appropriately.^12,15^ Resveratrol exerts its anti-OC effects by altering multiple molecular targets^16,17^ and regulating apoptotic and autophagic activities.^18^ For instance, the activated Wnt, Notch and STAT3 signaling pathways in human OVCAR-3 and CAOV-3 cells are concurrently inhibited, of which STAT3 inactivation seems a critical molecular event because selective inhibition of STAT3 signaling leads cancer cells to apoptosis.^19^ However, STAT3 signaling has repressive roles in autophagy of cancer cells with different biological consequences.^20^ For instance, STAT3 inhibits autophagy and pancreatic cancer cell growth by downregulating LC3 expression,^21^ whereas inhibition of this signaling suppresses growth and promotes autophagy and apoptosis of esophageal squamous cell carcinoma cells.^22^ These data suggest that the interplay of STAT3 signaling and autophagy, and its biological consequences to cancer cells, may vary by cell type. To date, no studies have addressed the role of STAT3 in regulating autophagic activity in OC cells and the impact of resveratrol on that process. The current study thus aims to address the above issues, using human OVCAR-3 and CAOV-3 as the experimental models because their growth and STAT3 activation can be concurrently suppressed by resveratrol.^19^

## Results

### Resveratrol suppressed OC cell growth

**Figure 1:**
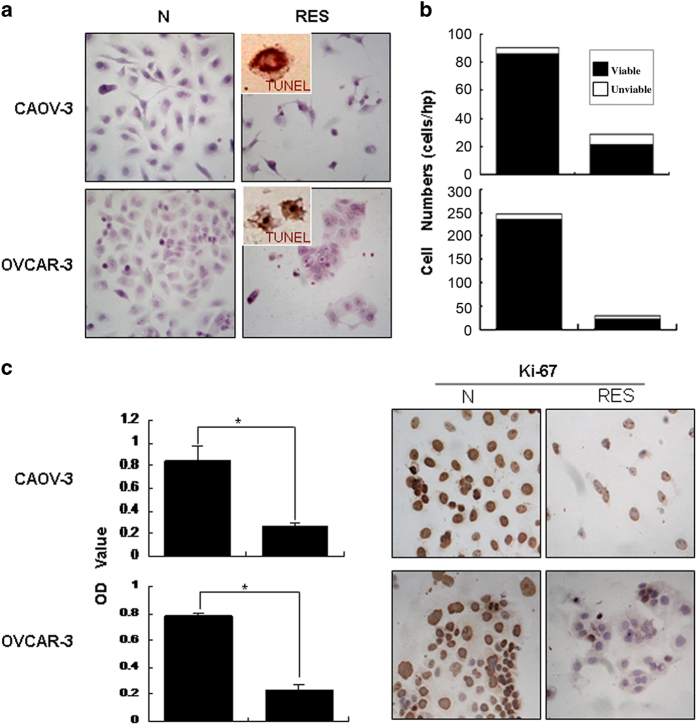
Cellular responses of human ovarian cancer CAOV-3 and OVCAR-3 cells to resveratrol. (**a**) Hematoxylin and eosin morphological staining and TUNEL staining performed on CAOV-3 and cells without (N) and with treatment with 120 *μ*M resveratrol for 48 h (RES). (**b**) Viable/unviable cell counting of CAOV-3 and OVCAR-3 cells treated by resveratrol for 48 h. (**c**) Evaluation of the responses of CAOV-3 and OVCAR-3 cells to resveratrol by MTT cell proliferation assay. (**d**) Ki-67-oriented immunocytochemical staining performed on CAOV-3 and OVCAR-3 cells without and with resveratrol treatment.

H/E staining showed distinct morphological alterations in resveratrol-treated populations (Figure 1a) and terminal deoxynucleotide transferase (TdT)-mediated dUTP-biotin nick-end labeling (TUNEL) assay demonstrated that TUNEL-positive cells appeared in the two cell lines after being treated by resveratrol for 24 h and becomes more popular at 48 h time point (Figure 1a). The viable/unviable cell fractionation and MTT assay revealed that the growth of all three OC cells was significantly suppressed (*P*<0.01) by 100 *μ*M resveratrol in time-related pattern in comparison with that of their normally cultured counterparts (Figures 1b and c). Ki-67-positive cells were greatly reduced after 48 h of resveratrol exposure (Figure 1d).

### Inactivated STAT3 signaling in resveratrol-suppressed OC cells

**Figure 2:**
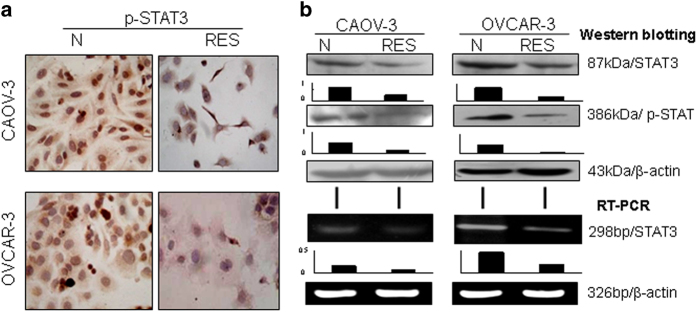
Statuses of STAT3 activation in CAOV-3 and OVCAR-3 cells without and with 120 *μ*M resveratrol treatment. (**a**) Immunocytochemical illustration of p-STAT3 intracellular distribution patterns in the two human ovarian cancer cell lines without (N) and with (RES) resveratrol treatment. (**b**) Western blot and RT-PCR analyses of STAT3 and p-STAT3 levels in the two human ovarian cancer cell lines without (N) and with (RES) resveratrol treatment.

Immunocytochemical staining demonstrated that p-STAT3 was distributed in either cytoplasm or the nuclei of the two OC cell lines, which became reduced in cytoplasm and rarely translocalized into nuclei after resveratrol treatment (Figure 2a). The results of western blotting were in agreement with ICC findings in terms of reduced level of p-STAT3 and the decreased production of total STAT3 proteins (Figure 2b). RT-PCR showed distinct downregulation of STAT3 transcription in resveratrol-treated OVCAR-3 and CAOV-3 cells (Figure 2c).

### Resveratrol-enhanced autophagic activities

**Figure 3:**
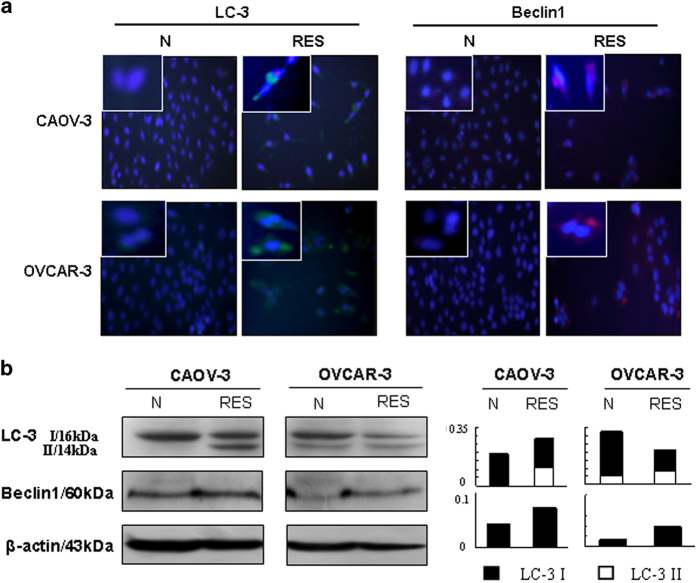
Evaluation of LC-3 and Beclin 1 expression in CAOV-3 and OVCAR-3 cells without (N) and with resveratrol treatment (RES) by immunofluorescent staining (**a**) and western blotting (**b**).

To elucidate the status of autophagy in OC cells and its relevance with resveratrol-caused cell crisis, double immunoflorescent staining for LC3 and Beclin-1 was conducted on the cell-bearing coverslips, which showed that these two proteins were weakly labeled in normally cultured OC cells and became enhanced especially for LC3 after being treated by 120 *μ*M resveratrol for 48 h (Figure 3a). The following H/E staining performed on the coverslips with double immunoflorescent staining revealed distinct cell death in resveratrol-treated populations (insets of Figure 3a).

### Resveratrol altered LC3 and Beclin-1 expression

The autophagic activities can be reflected by the levels of autophagy-associated protein LC3 and Beclin-1 and the presence of 14 kDa LC3-II, a proteolytic form of LC3.^23^ Therefore, the statuses of LC3 and Beclin-1 expression in the two OC cell lines without and with drug treatment were examined by western blotting. The results showed that the levels of LC3 and Beclin-1 were elevated in resveratrol-treated OC cells and an additional 14 kDa LC3-II band appeared in CAOV-3 and the increased fraction of this band in OVCAR-3 cells after 48 h of resveratrol treatment (Figure 3b).

### Negative correlation of STAT3 activation and Beclin-1 expression

Immunocytochemical staining with p-STAT3 antibody revealed that distinct nuclear translocation of STAT3 was commonly observed in normally cultured CAOV-3 and OVCAR-3 cells, which became weakened or diminished in the cells treated by 100 *μ*M resveratrol for 48 h (Figure 2a). In contrast, Beclin-1 was expressed in low levels in CAOV-3 and OVCAR-3 cells under normal culture condition and was upregulated upon resveratrol treatment (Figure 3b).

### LC3+/cytochrome C+ granules in resveratrol-treated cells

**Figure 4:**
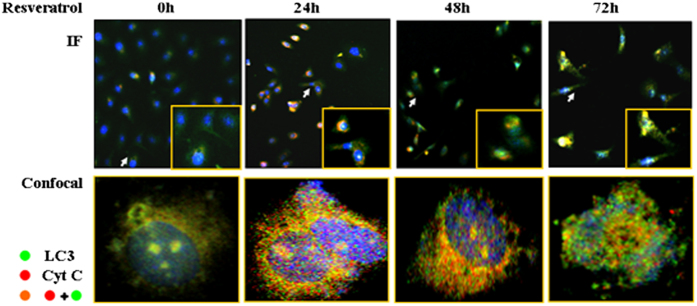
Double LC3 (green) and cytochrome C (red) immunofluorescent labeling of CAOV-3 cells treated by resveratrol at different time points. IF, the images taken under conventional IF microscope. Confocal, the images taken under confocal laser scanning microscope.

LC3 is a commonly used marker to monitor autophagosomes and cytochrome C proteins are preferably localized in mitochondria.^24^ To identify the feature(s) of autophagic bodies, LC3 and cytochrome C double IF staining combined with confocal laser scanning microscope observation were conducted on the OC cells without and with resveratrol treatment. The results demonstrate that the conventional IF image of normally cultured OC cells was weakly green in the cytoplasm and the confocal one showed the predominant distribution of granules labeled with LC3 (green) or cytochrome C (red; Figure 4a). The IF staining patterns were apparently altered in resveratrol-treated cells by showing the color transition of IF images from weakly green to strong orange and yellow at 24 h to green and yellow at 48 h and to yellow and green at 72 h time points. In agreement with the IF microscopic findings, the confocal images demonstrated the appearance of LC3^+^/cytochrome C^+^ granules in the cytoplasm of resveratrol-treated cells, especially those treated for 24 h (Figure 4b).

### AG490 induced autophagosis and apoptosis

**Figure 5:**
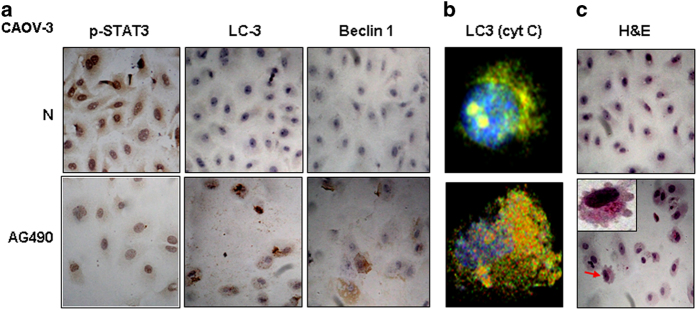
Evaluation of STAT3 activation, autophagic activities and apoptosis of CAOV-3 cells without (N) and with AG490 treatment (AG490). (**a**) Immunocytochemical staining for p-STAT3, LC-3 and Beclin 1; (**b**) Confocal images of double LC-3 and cytochrome C IF labeling; (**c**) H/E morphological staining performed on the IF stained coverslips for confocal examination. Arrow indicates the cell shown in the inset with higher magnification.

To elucidate the role of STAT3 signaling in regulating autophagic activity, AG490, a selective JAK2 inhibitor, was used to treat the CAOV-3 cell line for 48 h, followed by TUNEL apoptotic cell labeling, STAT3-oriented immunocytochemical staining and double immunoflorescent staining/IF for LC3/Becline-1 and LC3/cytochrome C. As shown in Figure 5, AG490 suppressed cell growth, inhibited STAT3 nuclear translocation and upregulated Becline-1 and, especially, LC3 expression. Furthermore, LC3+/cytochrome C+ granules (Figure 5c) and TUNEL-positive cells (Figure 5b) were frequently observed in AG490-treated CAOV-3 cells.

### Ultrastructural illustration of resveratrol increased autophagosomes

**Figure 6:**
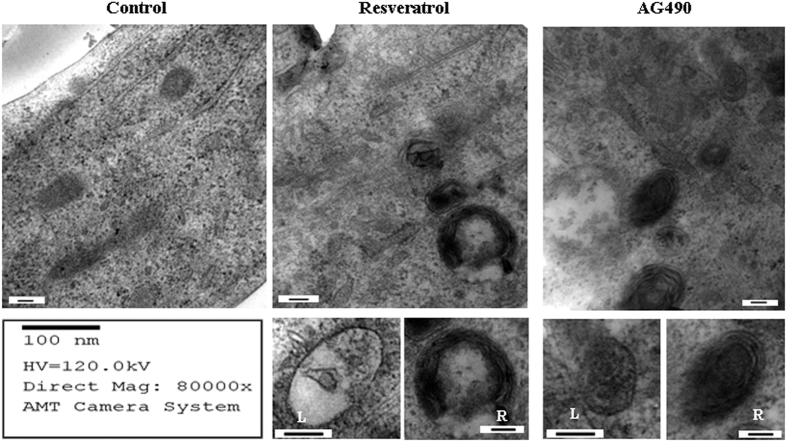
Electron microscopic illustration of autophagic statuses of CAOV-3 cells cultured normally (Control) and treated by resveratrol or AG490 for 48 h. The single membrane autophagosomes and the double membrane defined mitochondrial spheroids found in resveratrol- and AG490-treated cells are shown, respectively, with higher magnifications in the left (L) and the right (R) small images.

Electron microscopic graphs clearly demonstrated the abundance of cytosolic vacuoles enclosed with membrane bounded components in resveratrol- and AG490-treated OC cells rather than their normally cultured counterparts (Figure 6). It was also found that those vacuoles were not identical in terms of their sizes and contents (Figure 6b). As shown in the images in higher magnifications, some vacuoles were relatively small and involved the formation of a single outer membrane (left); in contrast, the others were larger, in a ring- or C-shaped morphology, defined by a double membrane and rich in criesta-like structures (right).

## Discussion

Increasing evidence demonstrates the promising inhibitory effects of resveratrol on OC cells.^19,25,26^ Our recent results show that Wnt, Notch and STAT3 signaling pathways are activated in human OC OVCAR-3 and CAOV-3 cells, which are concurrently inhibited by resveratrol, accompanied with remarkable growth arrest and cell death.^19^ Our previous study also indicates that STAT3 signaling is critical for the growth and survival of OC cells and is the major molecular target of resveratrol because STAT3 transcription is downregulated in resveratrol-treated OC cells. Further, the selective inhibition of STAT, but not the Wnt or Notch pathways, leads to a similar biological consequence caused by resveratrol, including the increased fractions of apoptosis.^19^ Nevertheless, it is still unclear whether apoptosis is the only death program triggered by resveratrol and inactivation of STAT3 signaling in OC cells, or whether additional survival or death mechanisms are altered as well.

Autophagy is a basic cellular maintenance mechanism by which unnecessary or dysfunctional cellular components are degraded and cellular survival can be maintained in response to stress.^27^ In the context of cancers, the roles of autophagy seem diverse; it occurs as an adaptive or protective response to environmental alterations,^28^ or it appears to commit the cells to programmed cell death type II.^29^ Although the relevance of autophagic activity with therapeutic efficacies of anticancer drugs has been discussed, the conclusions contradict. For instance, enhanced autophagocytosis has been found in resveratrol-sensitive OC cells,^18,30^ while it has a prosurvival role in resveratrol-induced cytotoxicity in human U251 glioblastoma cells.^31^ In agreement with Lang’s findings,^30^ we observed enhanced autophagic activity, together with distinct growth arrest and remarkable apoptosis, in OVCAR-3 and CAOV-3 cells treated by resveratrol for 48 h. In addition to the above cellular crisis, we found that resveratrol upregulates two autophagy-related genes, LC3 and Beclin-1. LC3 II generated by enzymatic cleavage indicates the initiation of autophagy.^23^ Our western blotting results clearly demonstrate the appearance of an additional LC3 II band and a twofold increase in the level of LC3II in resveratrol-treated OVCAR3 cells. These results suggest that resveratrol enhances autophagic activity of OC cells when exerting its anti-OC effects. Because the activated autophagocytosis is maintained throughout the treatment and is overlapped with apoptosis, this cellular event may reflect continuous turnover of intracellular components, which would be harmful for cell growth and maintenance.

STAT3 signaling is the critical survival factor of OC cells^19^ and the main molecular target of resveratrol.^12,19,32^ Although reduction of STAT3 expression can induce mitochondrial dysfunction and autophagy in mouse cardiac HL-1 cells,^33^ the involvement of this signaling pathway in regulating autophagic activities of cancer cells requires further study. Because of the co-existence of STAT3 inactivation and enhanced autophagic activity in resveratrol-treated OC cells, we elucidated the potential correlation of these two biological events by inhibiting STAT3 signaling with AG490, followed by LC3 immunocytochemistry staining. Similar to our findings from the resveratrol-treated cells, the nuclear translocation of p-STAT3 was largely inhibited, accompanied by enhanced LC3 and Beclin 1 expression and remarkable apoptotic cell death in AG490-treated OC cells. These findings thus indicate the importance of activated STAT3 signaling in regulating the autophagic activity of OC cells. Alternatively, the enhanced autophagic activity in resveratrol-treated OC cells may be, at least in part, the consequence of STAT3 inactivation.

Autophagocytosis occurs in several ways to degrade unnecessary or damaged intracellular elements. For instance, the autophagosomes that contain defective mitochondria are called mitochondrial autophagy or mitophagy.^34^ Although the enhanced autophagic activity has been found in resveratrol-treated OC cells with suppressed STAT3 signaling, it is worthwhile to know the type of autophagy happening in those cells because it may reflect the pattern and the extent of intracellular turnover caused by resveratrol and the STAT3 selective inhibitor. Cytochrome C, a small hemeprotein, appears on the surface of the inner mitochondrial membrane and acts as an essential component of the electron transport chain.^35^ Therefore, it can be used as a parameter to identify mitochondria.^36^ To determine the nature of LC3+ granules in resveratrol-treated OC cells and the cells treated by the STAT-selective inhibitor AG490, we performed double IF staining combined with confocal examination on those cells using a green-labeled mouse anti-human LC3 antibody and a red-labeled rabbit anti-human cytochrome C antibody. Merging of the LC3 and cytochrome C images changed many intracellular granules to orange-yellow, indicating they are positive for both LC3 and cytochrome C and contain a mitochondrial component. In contrast, LC3+/cytochrome C+ granules are uncommon in the OC cells cultured under normal conditions. Electron microscopic images further demonstrate the presence of two types of structural alterations of membrane-bounded compartments in resveratrol- or AG490-treated OC cells. One is defined by a single membrane and contains membrane elements in its lumen, the typical phenotype of autophagosome.^37^ The other is considered a mitochondrial spheroid because of its double membrane-surrounded ball-like structure with an internal compartment opening to the external cytoplasmic space.^38^ These phenomena suggest for the first time the abilities of resveratrol to increase overall autophagic activities and to cause extensive mitochondrial turnover. Because AG490-treated cells show the similar IF labeling pattern and ultrastructural phenotype, resveratrol-promoted autophagocytosis may be largely due to STAT3 inactivation.

Currently, the therapeutic implication of enhanced autophagic activity in cancer cells has not been well clarified.^39^ Because remarkable autophagocytosis overlaps with extensive apoptosis in resveratrol-treated OC cells, it is possible that resveratrol increases the pressure of intracellular damages, leading to enhanced autophagic elimination of the damaged elements, severe loss of organelle function and finally cell death.^40^ This supposition is supported by the presence of mitochondrial spheroid formations in resveratrol-treated OC cells, because this unique mitochondrial structure can be induced by mitochondrial toxin and evolved in the pathological conditions related to mitochondrial injury.^41^ So far, the relevance of mitochondrial spheroids with autophagy has not yet been ascertained. The findings of increased LC3+/cytochrome C+ granules and mitochondrial spheroids in resveratrol-suppressed OC cells indicate that the mitochondrial dysfunction and exhaustion may make OC cells more fragile to drug treatment. The existence of mitochondrial spheroids in AG490-treated cells implicates the importance of STAT signaling for maintaining mitochondrial integrity.^42^ However, this speculation should be further elucidated by determining whether the autophagic turnover and cell crisis can be rescued by prevention of resveratrol-caused STAT3 inactivation or reactivation of STAT3 downstream signaling in resveratrol-treated OC cells.

Taken together, our current study demonstrates resveratrol’s abilities to promote autophagy and apoptosis and to cause mitochondrial turnover in human OC cells. This further confirms the therapeutic value of this nontoxic compound in the adjuvant management of OCs. The extremely low bioavailability of orally administered resveratrol has long been the major obstacle to clinical use of the compound. According to our findings from rat orthotopic glioblastoma and bladder cancer models,^12,15^ this can be largely overcome by organ-oriented administration approaches. In this context, intraperitoneal injection, a conventional way to treat abdominal malignancies,^43^ would be a potential option for practical treatment of OCs with resveratrol.

## Conclusions

This study demonstrates the abilities of resveratrol to promote autophagic and apoptotic activities and to inactivate STAT3 signaling in human OC cells. This nontoxic compound would therefore be of therapeutic value in the adjuvant management of OCs when administered appropriately.

## Materials and methods

### Cell culture and treatment

Human OC CAOV-3 cells^44^ were cultured in Dulbecco’s modified Eagle’s essential medium containing 12% fetal bovine serum (Gibco Life Science, Grand Island, NY, USA) under 37 °C and 5% CO_2_ conditions and human OC OVCAR-3 cells^45^ in RPMI1640 under 37 °C and 5% CO_2_ condition. The cells (5×10^4^/ml) were plated to culture dishes (NUNC, Denmark) and incubated for 24 h before the experiments. *Trans*-resveratrol (Sigma Chemical Co., St. Louis, MO, USA) was dissolved in DMSO (Sigma) to a stock concentration of 100 mM, wrapped in aluminum foil for protection against light and stored at −20 °C. The cells were exposed to 120 *μ*M Res for 72 h (72 h). The cells cultured under normal condition and treated by 0.2% DMSO were used as normal and background controls, respectively. Cell numbers and viabilities were checked in 24 h intervals. The cell-bearing coverslips collected from the three experimental groups were fixed in cold acetone or 4% paraformaldehyde (pH 7.4) for morphological and immunocytochemical examinations. The experimental groups were set in triplicate and the experiments were repeated at least for three times to establish confidential conclusion.

### Evaluation of cell growth

Haematoxylin and eosin (H/E) staining was performed on the cell-bearing coverslips to evaluate the morphological features of the three OC cell lines with different treatments. The effects of resveratrol on cell proliferation were determined by 3-[4,5-Dimethylthiazol-2-yl]-2,5-diphenyl-tetrazolium bromide (MTT) assay^17^ and shown in OD values. The fractions of viable and unviable cells were estimated with cell counting apparatus (TC20 Automated Cell Counter, BIO RAD). TUNEL assay was employed to detect apoptotic cells according to producer’s instructions (Promega Corporation, USA).

### Sequential coverslip collection from resveratrol-treated populations

It has been proposed that altered autophagic activity is the early cellular response to drug treatment^46^ and has certain relevance with chemosensitivities of the treated cells.^46–48^ NEST-DISH, a joint product of NEST Biotech Inc. Wuxi and BioArrayTech Inc. Dalian, China (China Patent for Invention: ZL200610047607.0), is the novel coverslip preparation dish, from which 32 cell-bearing coverslips can be prepared and then collected sequentially or simultaneously for different research purposes.^49^ Therefore, this novel device was adopted for sequential analyses of the statuses of autophagic activities and STAT3 signaling of resveratrol-treated cells by collecting several pieces of cell-bearing coverslips at 4 time points (0, 24, 48 and 72 h) of resveratrol treatment. The coverslips collected were fixed properly for H/E morphological staining, LC3 and Becline-1 double immunofluorescent staining, STAT3-oriented immunocytochemical staining and, when necessary, TUNEL apoptotic cell labeling.

### Immunocytochemical staining

Immunocytochemical staining (ICC) was performed on the cell-bearing coverslips collected regularly from different experimental groups by the method described previously.^18^ The antibodies against human STAT3 and p-STAT3 were purchased from Santa Cruz Biotechnology, Inc, CA. Color reaction was developed using 3, 3′-diaminobenzidine tetrahydrochloride (DAB). According to the labeling intensity, the staining results were evaluated by two independent researchers and scored as negative (−) if no immunolabeling was observed in target cells, weakly positive (+) if the labeling was faint, moderately positive (++), and strongly positive (>++) when the labeling was stronger or distinctly stronger than (++).

### Immunofluorescent labeling and observation

For immunofluorescent staining (IF), the cell-bearing coverslips were rinsed with phosphate-buffered solution (PBS; pH 7.4) for 3 times, blocked with 10% goat serum in PBS (pH 7.4) for 20 min, incubated overnight with primary antibody against target protein (LC3 or Becline-1) and finally co-incubated with fluorescence-labeled goat anti-rabbit and rabbit anti-mouse IgG (1 : 200; Santa Cruz Biotechnology, Inc) at 37 °C for 60 min in darkness. The nuclei were labeled by DAPI (blue fluorescence). The coverslips were observed and photographed under a fluorescence microscope (BX51, Olympus, Japan) and a confocal laser scanning fluorescent microscopy (SP8, Leica, Heidelberg, Germany).

### RNA isolation and RT-PCR

Sample RNAs were isolated from the two human OC cell lines without and with resveratrol treatment for 48 h. By the method described elsewhere,^8^ reverse transcription (RT) was performed on RNA samples, followed by polymerase chain reaction (PCR) with a pair of primers specific for the cDNA of an individual gene (STAT3: Forward primer: 5′-GGGTGGAGAAGGACATCAGCGGTAA-3′, reverse primer: 5′-GCCGACAATACTTTCCGAATGC-3′; *β*-actin: forward primer: 5′-GCATGGAGTCCTGTGGCAT-3′, reverse primer: 5′-CTAGAAGCA TTTGCGGTGG-3′^19^). The PCR products were resolved on 1% agarose gel containing ethidium bromide (0.5 *μ*g/ml), visualized and photographed using UVP Biospectrum Imaging System (UVP, Inc, Upland, CA). The *β*-actin PCR products generated from the same RT solution were cited as quantitative controls.

### Protein preparation and western blotting

Total cellular proteins were prepared from the cells under different culture conditions. The sample proteins (50 *μ*g/well) were separated in 10% sodium dodecylsulfate-polyacrylamide gel electrophoresis and transferred to polyvinylidene difluoride membrane (Amersham, Buckinghamshire, UK). The membrane was blocked with 5% skimmed milk in TBS-T (10 mM Tris-HCl, pH8.0, 150 mM NaCl and 0.5% Tween 20) at 4 °C, rinsed 10 min for three times with TBS-T, followed by 3 h incubation at room temperature with the first antibody and then 1 h incubation with HRP-conjugated anti-mouse or anti-rabbit IgG (Zymed Lab, Inc). The bound antibody was detected using the enhanced chemiluminescence system (Roche GmbH, Mannheim, Germany). After removing the labeling signal by incubation with stripping buffer,^8^ the membrane was reprobed with other antibodies one by one until all of the parameters were examined.

### Autophagosome identification and quantification

LC3 acts downstream from the ATG5-ATG12 system and distributes on the outer and inner surfaces of the autophagosome.^50^ Cytochrome C, a component of the electron transport chain, is found loosely associated with the inner membrane of the mitochondrion.^35^ Therefore, these two proteins were employed as the biomarkers for identifying the nature(s) of the increased autophagosomes in resveratrol-treated OC cells by double IF staining. The criteria of the judgment were as follows: the intracellular LC3-positive green granules under fluorescent microscope indicated the presence of autophagosomes; the cytochrome C-positive red granules indicated that they were mitochondria or contained mitochondrial elements; the granules co-labeled by LC3 and Cytochrome C suggested that they were mitochondrial autophagosomes. The LC3 and Cytochrome C labeling patterns of OC cells without and with resveratrol or AG490 treatment were visualized in the forms of conventional IF microscopic and confocal IF microscopic images.

### Treatment with AG490 selective JAK2 inhibitor

AG490 has been regarded an upstream STAT inhibitor.^51^ To elucidate the influence of STAT inhibition in autophagic activity, this compound in the working concentration of 80 *μ*M was therefore used to treat the three OC cells growing on the coverslips for 48 h. The coverslips collected were subjected to H&E morphological staining, Beclin-1 and p-STAT3 immunocytochemical staining and double immunoflorescent staining using rabbit anti-human LC3- and mouse anti-human cytochrome C antibodies.

### Electron microscopic examination

The cell-bearing coverslips were washed with PBS for three times (10 min/time) and then fixed in 2.5% glutaraldehyde (30 min, 50 mM cacodylate buffer, pH 7.2) and 2% OsO_4_ (30 mim, same buffer). Ultra-thin sections (0.1 *μ*M) were prepared and examined under a Philips CM100 transmission electron microscope (FEI Company, USA). Images were captured by charge-coupled device camera equipped with TCL-EM-Menu version 3 from Tietz Video and Image Processing Systems (Gaunting, GmbH, Friedrichshafen, Germany) as described elsewhere.^29^ The coverslips bearing the cells without resveratrol or AG490 treatment were used as controls.

### Statistical analysis

Statistical analyses were performed using the software of SPSS 17.0 (IBM, San Francisco, CA, USA). Data were given as the means±S.D. Statistical analyses were performed using one-way ANOVA and *P*<0.05 were considered to indicate significance.
